# Vascular Diseases in Women: Do Women Suffer from Them Differently?

**DOI:** 10.3390/jcm13041108

**Published:** 2024-02-15

**Authors:** Katalin Farkas, Agata Stanek, Stephanie Zbinden, Barbara Borea, Simina Ciurica, Vanessa Moore, Peggy Maguire, Maria Teresa B. Abola, Elaine B. Alajar, Antonella Marcoccia, Dilek Erer, Ana I. Casanegra, Hiva Sharebiani, Muriel Sprynger, Maryam Kavousi, Mariella Catalano

**Affiliations:** 1 Department of Angiology, Szent Imre University Teaching Hospital, Tétényi út 12-16, 1115 Budapest, Hungary; 2 VAS-European Independent Foundation in Angiology/Vascular Medicine, Via GB Grassi 74, 20157 Milan, Italy; astanek@tlen.pl (A.S.); stephanie.zbinden@usz.ch (S.Z.); barbaraborea@hotmail.it (B.B.); simina.ciurica@humani.be (S.C.); mbabola@up.edu.ph (M.T.B.A.); antonella.marcoccia@aslroma2.it (A.M.); dilekerer@gazi.edu.tr (D.E.); hivasharebiani@yahoo.com (H.S.); msprynger@chuliege.be (M.S.); m.kavousi@erasmusmc.nl (M.K.); mariella.catalano@unimi.it (M.C.); 3 Department of Internal Medicine, Angiology and Physical Medicine, Faculty of Medical Sciences in Zabrze, Medical University of Silesia, Batorego 15 Street, 41-902 Bytom, Poland; 4 Department of Angiology, Zurich University Hospital, Ramistrasse 100, 8091 Zurich, Switzerland; 5 Department of Angiology and Haemostasis, Geneva University Hospitals, Rue Gabrielle-Perret-Gentil 4, 1205 Genève, Switzerland; 6 Department of Cardiology, Marie Curie Civil Hospital, CHU Charleroi, Chaussée de Bruxelles 140, 6042 Lodelinsart, Belgium; 7 European Institute of Women’s Health, Ashgrove House, Kill Avenue, Dún Laoghaire, A96 N9K0 Dublin, Ireland; moorev@tcd.ie (V.M.); peg@eurohealth.ie (P.M.); 8 Clinical Research Department, Education, Training and Research Services, Philippine Heart Center, University of the Philippines College of Medicine, 547 Pedro Gil Street, Manila 1000, Metro Manila, Philippines; 9 Manila Doctors Hospital, 667 United Nations Ave, Ermita, Manila 1000, Metro Manila, Philippines; ebalajar@up.edu.ph; 10 Angiology and Autoimmunity Medical Unit, Rare Diseases Reference Center for Systemic Sclerosis, Sandro Pertini Hospital, 00157 Rome, Italy; 11 Gazi University Hospital, Mevlana Blv. No:29, Yenimahalle, Ankara 06560, Turkey; 12 Gonda Vascular Center, Department of Cardiovascular Medicine, Mayo Clinic, 200 1st Street SW, Rochester, MN 55901, USA; casanegra.ana@mayo.edu; 13 Support Association of Patients of Buerger’s Disease, Buerger’s Disease NGO, Mashhad 9183785195, Iran; 14 Department of Cardiology, University Hospital of Liège, Hospital Boulevard, 4000 Liege, Belgium; 15 Department of Epidemiology, Erasmus MC, University Medical Center Rotterdam, Dr. Molewaterplein 40, 3015 GD Rotterdam, The Netherlands; 16 Department of Biomedical and Clinical Science, Inter-University Research Center on Vascular Disease, University of Milan, GB Grassi 74, 20157 Milan, Italy

**Keywords:** women, vascular diseases, risk factors, gender differences

## Abstract

According to the World Health Organization, cardiovascular disease (CVD) is the leading cause of death among women worldwide, yet its magnitude is often underestimated. Biological and gender differences affect health, diagnosis, and healthcare in numerous ways. The lack of sex and gender awareness in health research and healthcare is an ongoing issue that affects not only research but also treatment and outcomes. The importance of recognizing the impacts of both sex and gender on health and of knowing the differences between the two in healthcare is beginning to gain ground. There is more appreciation of the roles that biological differences (sex) and sociocultural power structures (gender) have, and both sex and gender affect health behavior, the development of diseases, their diagnosis, management, and the long-term effects of an illness. An important issue is the knowledge and awareness of women about vascular diseases. The risk of cardiovascular events is drastically underestimated by women themselves, as well as by those around them. The purpose of this review is to draw attention to improving the medical care and treatment of women with vascular diseases.

## 1. Introduction

Vascular diseases (arterial, venous, lymphatic, and microcirculatory) have a considerable weight worldwide. All vascular patients suffer from a lack of diagnosis, specialist offers, and equity [1], and the latter becomes more severe in women. This review focuses on vascular diseases in women in terms of relevance and the need for a common position coordinated with the valuable existing international actions on women’s health [2]. Main risk factors, as well as main vascular diseases, are evaluated considering gender specificity.

Key points to consider in women’s health are related to the disease as well as to the availability of health education and carriers. They have a general value described as follows:

1. Differences in physiopathology exist between the genders, asking for more integrated and different approaches [3].
2. Epidemiological data show differences in distribution, deterioration, and outcomes to be detailed and considered [4].
3. Similar diseases have different symptoms in women, which are often under-evaluated [2]. A delay in visiting the doctor that is linked to gender has been described.
4. Behaviors associated with gender also involve exposure to risk factors and attitudes to disease prevention and response.
5. Some diagnostic parameters should be validated to verify any possible differences related to gender.
6. Women’s responses to therapy have been reported to be sometimes different. There is a need for proper studies and validation in women. Although women’s enrollment in clinical trials is low, the conclusions are considered valid without any gender distinctions [5].
7. Health is linked to social determinants [6,7], and some of these are specific or have a greater weight for women.
8. There is a need to guarantee equal access, development, and opportunities for women to get the best qualifications, gain work in the health area, and become doctors, researchers, and academics. Gender equality in science, medicine, and health contributes to gender equality in the community and gives social benefits. The UN Educational, Scientific and Cultural Organization’s Women in Science data show that, even with a recent improvement, less than 30% of the world’s researchers are women, with relevant geographical differences [8].

Each area of medicine can contribute to highlighting its specific aspect, to enforce recommendations and action to overcome the existing gap in gender equity.

## 2. Cardiovascular Risk Factors in Women

As per the World Health Organization, cardiovascular disease (CVD) stands as the foremost contributor to mortality in women globally, yet its magnitude is often underestimated. Most CVD cases can be prevented by identifying and managing modifiable cardiovascular risk factors (CVRF). There are CVRF that are common to both sexes, with specificities, and others that are unique to women.

### 2.1. Conventional Cardiovascular Risk Factors in Women

Aging remains an important CVRF; however, compared to men, women present with CVD about 10 years later, probably because of reduced CVRF exposure [9].

Other major CVRF—hypertension (HTN), diabetes mellitus (DM), low physical activity, and moderate alcohol consumption—exhibited a stronger association with myocardial infarction (MI) in women, as highlighted by the INTERHEART study [10]. Furthermore, a recent large-scale meta-analysis involving more than 5 million patients indicated that diabetic women present a 30% significantly higher risk of CVD mortality and 58% increased risk of coronary artery disease (CAD) compared to men [11]. Data on sex-specific links between HTN and CVD risk are conflicting. HTN seems to progress more rapidly in women compared to men, starting in the third decade of life, exposing women to a higher risk of developing MI [9,12]. However, a large-scale meta-analysis failed to show significant sex differences in the overall cardiovascular risk associated with HTN [13].

Female smokers face an increased risk of CAD, and their risk of developing MI is twice that of equivalent men [14,15]. In addition, smoking exposes women to an almost 30 times greater risk of abdominal aortic aneurysm (AAA) and puts them at a higher risk of developing AAA compared to men who never smoke [16]. These findings raise questions about the current European screening guidelines that do not recommend population screening in women (Class III level B), regardless of their smoking status [17].

The SWAN study revealed dramatic increases in total cholesterol, LDL-C, and (Apo)B levels related to menopause that correlate with the presence of long-term carotid plaques [18]. In secondary prevention, women’s lipid levels remain higher, with only 51% of women achieving dyslipidemia control compared to 63% of men, differences likely attributable to lifestyle factors, adherence to medication, and physical activity [19].

Obesity raises the probability of developing ischemic heart disease by 64% in women versus 46% in men, as demonstrated in The Framingham Heart Study [20]. Moreover, according to a large meta-analysis, women require higher levels of physical training to decrease their risk of stroke [21].

Other than the classical CVRF, depression [22], trauma exposure [23], and socioeconomic status [24] are being increasingly recognised as risk factors for CAD in women, probably impacting CV status via unhealthy behaviors and by accelerating other CVRF that result in rapid progression of atherosclerosis, although other mechanisms such as inflammation or autonomic nervous system dysfunction have been suggested [25].

### 2.2. Menopause-Related Cardiovascular Risk Factors

Endogenous estrogen seems to impart a protective effect against CVD, leading to a lower cardiovascular risk compared to men of the same age [26]. This protective influence operates by mitigating vascular damage, via anti-inflammatory and immunomodulatory activity, which is achieved by reducing the expression of pro-inflammatory mediators and increasing the production and release of nitric oxide (NO). This, in turn, results in a decrease in C-reactive protein levels in women of reproductive age. Interestingly, this stands in contrast to exogenous estrogen, which has been observed to increase C-reactive protein levels [27].

If endogenous estrogens exhibit a CV-protective effect in premenopausal women compared to men, the incidence of CVD drastically increases after menopause. Postmenopausal women face a CVD incidence up to six times greater than that of premenopausal women. Moreover, complications associated with CVD exceed those of men [28]. Estrogen deficiency emerges as the primary factor doubling cardiovascular morbidity and mortality among women. Despite this, the mechanisms contributing to this increase remain unclear [29].

The transition to menopause, marked by estrogen deficiency, has a profound impact on cholesterol metabolism, redistribution of body fat toward abdominal and subcutaneous regions, increased insulin resistance, and vascular endothelial dysfunction. This transition is associated with reduced endothelial cell turnover and reduced production of vasoactive, anti-inflammatory and antiatherogenic compounds, ultimately leading to increased atherosclerotic plaque formation [30].

The analysis, based on pooled individual data from ten observational studies, examined the associations between the type of menopause, age at menopause, and the risk of the first non-fatal cardiovascular manifestation in more than 200,000 postmenopausal women [31]. The results showed a graded relationship for cardiovascular disease (CVD) incidence in younger menopausal women for both natural and surgical menopause. Additionally, a significant association was found between the type of menopause, age at menopause, and the risk of CVD. Specifically, women with surgical menopause under the age of 35 and between 35–39 years old exhibited a higher risk of CVD compared to women with natural menopause aged 50–54 years. It is important to note that gynecological indications for the surgery were not available, and fatal CVD events were excluded from the analysis.

Although shorter durations of estrogen exposure and the absence of estrogen’s protective CV effects during the reproductive lifespan, as observed in premature menopause (PM) and early menopause (EM), are associated with an increased risk of CVD, when compared to smoking, hormone therapy, or Type 2 diabetes mellitus (Type 2 DM), PM and EM represent moderate yet significant risk factors for CVD. In these women, adopting a healthy lifestyle remains crucial for CVD prevention. However, more research is needed to determine whether these results are independent of menopausal hormone therapy (MHT), which can be a major confounder in the association between PM/EM and CVD [32].

The use of MHT after menopause for the prevention of cardiovascular disease (CVD) is a topic of controversy. MHT appears to offer protection against CVD in younger postmenopausal women, but no significant effects on CVD outcomes are found 10 years after menopause [33]. The International Menopause Society and the American Heart Association recommend MHT for CV prevention shortly after menopause, particularly in perimenopausal or postmenopausal women under 60, but not in women over 60 or 10 years after menopause [34,35]. Numerous post-hoc analyses conducted on the Women’s Health Initiative (WHI) data provide support for the “timing hypothesis”. This hypothesis suggests that the vasoprotective effects of hormones diminish in elderly women. Interestingly, some studies suggest that MHT might increase the relative risk of CAD compared to a control group during the first 2 years of treatment, but this risk diminishes between 3 and 7 years [36]. A Cochrane review [37] assessed the long-term effects of MHT on mortality, cardiovascular outcomes, cancer, fractures, and quality of life and showed that MHT is not indicated for primary or secondary prevention of CVD in postmenopausal women. However, it demonstrated benefit for the prevention of postmenopausal osteoporosis.

### 2.3. Pregnancy and Reproductive Risk Factors

Early menarche, defined as occurring before 10 years of age, is associated with elevated waist circumference, HTN, obesity, hyperlipidemia (HLD), DM, and a 15% to 30% higher risk of CVD in adulthood. Mechanisms connecting EM to elevated CVD are incompletely understood. After adjusting for age, physical activity, smoking, alcohol, educational level, occupational social class, oral contraceptive use, hormone replacement therapy, parity, BMI, and waist circumference, the estimates of CVD risk were slightly reduced; however, the risk remained significant [38,39].

Polycystic ovary syndrome (PCOS) is associated with various CVRF, such as impaired glucose tolerance, HLD, HTN, metabolic syndrome, and Type 2 DM. Moreover, previous studies have reported an increased likelihood of elevated subclinical markers of CVD in women with PCOS, including coronary artery calcium, C-reactive protein levels, and carotid intima-media thickness [40]. However, the status of PCOS as an independent CVRF for CVD remains unclear [41,42].

Hypertensive disorders of pregnancy (HDP) include chronic HTN before pregnancy, gestational HTN, and preeclampsia/eclampsia [40]. These disorders pose significant risks for both short- and long-term CVD. During the peripartum period, women with a history of HDP have significantly higher odds of experiencing stroke, myocardial infarction, cardiomyopathy, and spontaneous coronary artery dissection compared to those without HDP. It is also well-established that this increased risk of CVD extends well into later life. [43,44]. A meta-analysis has established a twofold risk of CVD among women with a history of gestational HTN or preeclampsia (RR 2.50 [95% CI, 1.43–4.37]). [44]. The association between HDP and CVD is attenuated when adjusted for other CVRF but persists. The association between HDP and CVD are poorly understood [43].

About one-third of women experiencing gestational diabetes mellitus (GDM) will develop Type 2 DM with time, which poses a 7-fold higher risk compared to women without GDM. [45]. Women diagnosed with GDM appear to have twice the risk of CVD in the future compared to those without GDM (RR 1.98 [95% CI, 1.57–2.50]) [46].

An increasing number of women are turning to fertility therapy; these individuals are generally older and often have more comorbidities than younger women. Studies investigating an increased risk of CVD after fertility treatment show conflicting results, and a clear causality has not been established [47]. One of the complications is ovarian hyperstimulation syndrome, which leads to thromboses, renin angiotensin system activation, and vascular injuries. Prevention of this syndrome is crucial. More studies are needed on treatments and prevention strategies [48,49].

Pregnancy itself is a cardiovascular risk factor. Being a current smoker during pregnancy has been demonstrated to contribute cumulatively to the risk of myocardial infarction [50].

A post-hoc study by WHI found that women who breastfed for a duration exceeding 12 months during their lifetime had a reduced risk of developing HTN (odds ratio [OR] 0.88, *p* < 0.001), DM (OR 0.80, *p* < 0.001), HLD (OR 0.81, *p* < 0.001), or CVD (OR 0.91, *p* = 0.008) in comparison to women who never breastfed [51].

Sex-specific risk factors are vital for assessing comprehensive CVD risk in women. Further research is needed to understand their pathophysiology and mechanistic links to later-life CVD, informing precision prevention strategies. Considering the specificities of women is essential for effectively managing conventional CVRF.

## 3. Gender Differences in Health, Gender, and Clinical Trials

Biological and gender differences affect health, diagnosis, and healthcare in numerous ways. Traditionally, in medicine and healthcare the male body was considered the norm and women, the female body, and its symptoms were considered deviations from this norm, with the major exception being sexual/reproductive diseases. Also, traditionally women have been and largely still are underrepresented in clinical trials, even when they concern diseases that occur predominantly in women [52]. Much of the underrepresentation of women in clinical trials can be dated back to the thalidomide tragedy, where women were given medication for morning sickness in pregnancy in the belief that the medication was safe—however, it resulted in severe birth defects. The testing standards were very different in the 1950s, and the drug had primarily been tested on mice, which were less sensitive to thalidomide than other species [53]; also, thalidomide was a drug that had been shown to be minimally harmful to adults, but that had a very high degree of embryotoxicity [54]. After this, due to perceived risk, women of reproductive age were actively discouraged from participating in clinical trials [55], and although such discouragement is no longer a policy, women continue to be underrepresented in clinical trials.

The lack of sex and gender awareness in health research and healthcare is an ongoing issue that affects not only research, but also treatment and outcomes. Even basic awareness of the concepts of sex and gender is limited. Sex and gender are two separate terms that both impact on health; however, they tend to be conflated in medicine. A 2022 study of biomedical publications found that the term ‘gender differences’ only analyzed and reported biological occurrences, demonstrating how such terminology is still used interchangeably and incorrectly in medical research [56]. Sex refers to characteristics that are biological and physiological that define men and women, whereas gender describes socially constructed characteristics, norms, and behaviors that supposedly characterize women and men [57].

Furthermore, gender exists in a spectrum and can also be broken down into a number of categories, for example, gender identity, gender relationships, etc. [58]. Additionally, to fully understand the impact of gender, it is important to recognize its intersection with other inequalities, and how interactions between gender, race, and groupings, for example, sexuality, physical or mental disability, age, and so on, effect outcomes, experiences, and power structures [59]. All of these issues affect a person’s health, especially social determinants of health.

Failures to address sex and gender as important variables in cardiovascular research as well as in reporting, and the resulting limited understanding of differences based on both sex and gender, have contributed to the existing disparities in cardiovascular care and outcomes [60]. This understanding can have grave effects on women and their health outcomes, both in cardiovascular disease and in other diseases. With cardiovascular disease, clinical guidelines have been based on data that predominantly included male study participants, and then being generalized to fit women, regardless of actual evidence of this being correct. Nicholas and colleagues describe a telling example in which a clinical trial of heart failure medications (i.e., beta-blockers, angiotensin-converting enzyme inhibitors, or angiotensin receptor blockers) proposed that half the target dose created the best response in women, and that women experienced more of a risk of side effects when taking a higher dose. However, no prescription doses adjusted for sex have been established [61]. This example, which is only one among numerous, shows that insufficient female participation in clinical trials leads to significant issues, including inclusion criteria dominated by the male standard, outcome measurements that are sex-biased, inadequate data analysis, and a failure to transfer research results into clinical practice [62], but even when differences are known, these are not always applied in practice.

There are some recent improvements that give reason for optimism in relation to increased female participation in clinical trials. The EU Clinical Trial Regulation No 536/2014 has improved clinical trial data transparency and specifies that the subjects participating in a clinical trial should represent the population groups, such as gender or age groups, that are likely to use the medicinal product. It also contains provisions for including pregnant and breastfeeding women in clinical trials, as well as a breakdown of data by sex and gender [63]. Sosinsky et al. found that female representation in early-phase research has improved in cardiovascular clinical trials, which is good news as women have been especially underrepresented in early-phase clinical trials. However, they also found that although approximately 49% of patients with cardiovascular disease are women in the US, the average trial included only 41.9% women. As the US has already mandated in 1993 the adequate inclusion of women in trials sponsored by the National Institutes of Health (NIH) to determine sex differences, and has established the FDA’s Office of Women’s Health in 1994 [52], they are decades ahead of Europe in efforts to implement increased female participation in clinical trials but are still finding that women are underrepresented. Moreover, if women have been included in clinical trials, the results are generally not disaggregated or analyzed for gender differences. Unless such a systematic analysis is mandatory, the future looks bleak for women.

The importance of recognizing the impact of both sex and gender on health and of knowing the differences between the two in healthcare is beginning to gain ground. There is a greater appreciation of the role biological differences (sex) and sociocultural power structures (gender) play, and both sex and gender affect health behavior, the development of diseases, their diagnosis, management, and long-term effects of an illness [58]. However, there is still a long way to go and there is still a continuous need to advocate and highlight the need for gender and sex awareness in health and research, and of the underrepresentation of women in clinical trials.

## 4. Vascular Diseases in Women

### 4.1. Cerebrovascular Disease

The WHO reports that, globally, there are more than 77 million people who are currently living and have had a stroke, and almost two-thirds of these were due to ischemic stroke. Annually, 55% of all ischemic strokes occur in women and fifty-two percent of all stroke-related deaths are among women [64]. One of the important risk factors for stroke incidence and mortality is advanced age. Men have a higher lifetime risk of ischemic stroke but, beyond the age of 85, stroke risk is higher in women [65]. Elderly women have more severe strokes, poorer outcomes, and more difficult access to stroke care [66]. Beyond the age of 60, changes in vascular function, such as endothelial dysfunction and arterial stiffness, are accelerated in women [67].

Atherosclerotic carotid artery disease is one of the treatable causes of cerebrovascular disease. In 1999, the Tromsø study, which was among the first to describe sex differences in the prevalence of atherosclerotic carotid artery disease, reported that more women beyond the age of 75 had more carotid plaques [68]. More contemporary data on women aged 30–79 years report that the estimated prevalence rates of increased carotid intima-media thickness, carotid plaque, and carotid stenosis are lower among women versus men: 23.2% vs. 32.1%, 17.1% vs. 25.2%, and 1.2% vs. 1.8%, respectively [69].

Sex differences in carotid plaque morphology and composition based on histology and non-invasive imaging have been reported. Women were found to have higher rates of stable fibrous/fibrocalcific plaques, while men had higher rates of unstable plaques, with high-risk features such as large intraplaque hemorrhage, thin fibrous cap, large lipid core, and more inflammatory cells [70].

The role of sex hormones in the development of atherosclerosis, plaque instability, and stroke risk has been reported. Estradiol appears to have a protective effect in women against stroke that decreases after menopause [71]. Thus, it is believed that estradiol can slow the progression of atherosclerosis if hormone therapy is started soon after menopause when the endothelium is relatively healthier [72].

Sex differences in the management of carotid disease have also been described, with a greater reduction in stroke risk observed in women when medical management was offered in large trials of carotid stenosis trials; however, women were reported to be less likely to receive preventive medical therapy. On the other hand, women were underrepresented in carotid surgery trials and only post-hoc analyses suggested that women benefited less from surgical intervention, although medical management at that time was not as optimal as what current medical treatment can offer. Furthermore, postoperative outcomes have been reported to be less favorable among women and it has been hypothesized that this may be due to smaller mean diameters of the carotid arteries in women, which may present greater technical difficulty during carotid endarterectomy [16].

### 4.2. Abdominal Aortic Aneurysms

Gender-based differences in prevalence, diagnosis and management have been reported in abdominal aortic aneurysms (AAA). In a cross-sectional study of 1.5 million women and 0.8 million men tested in clinics, the overall prevalence of AAA was 0.6%, and it more common in men (1.5%) compared to women (0.25%), with higher prevalence in smokers. Compared to nonsmokers, the risk of AAA among those with a smoking history was 15 times greater in women (RR 15, 13.2–17.0), with significant associations in women <75 years (RR 26.4, 20.3–34.2) [73].

The 2022 AHA/SVS [58] and 2019 [17] ESVS Guidelines recommend repair in unruptured aneurysms with a diameter of 5.0 cm in women, with recommendation levels of class IA and class IIB, respectively. In a Swedish registry of 32,393 AAA patients, the proportion of treated versus untreated women was lower (17% versus 23%), and treated women were older compared to men (76.1 [8.5] years versus 73.0 [8.5] years) and more frequently suffered from COPD. Although the median times to rupture and death were similar at approximately 2.8 years, there was a higher risk of rupture and poorer survival among untreated female patients at all time points up to 5 years after diagnosis [74]. In a retrospective cohort of 16,386 patients with AAA, surgical repair was performed in 27% of women compared to 18% of men (*p* < 0.001). Women were more likely to undergo open surgical repair (RR 1.65; 95% CI 1.51–1.80), with open repair indicated for elective (RR, 1.82, 95% CI, 1.65–1.99) and symptomatic (RR, 1.46, 95% CI, 1.15–1.81) aneurysm. In those who had endovascular repair, women were more likely to have traditional risk factors such as smoking, older age, hypertension, and a family history of AAA. A similar trend was observed with open repair.

Women presented with smaller aneurysms (mean [SD], 57 [11.7] mm vs. 59 [17.7] mm in men; *p* < 0.001). Among those where aortic anatomy data was collected during endovascular repair (EVR), aortic neck lengths and neck diameters were shorter while aorta-neck angles and neck-AAA angle were larger in women. Between 2003 and 2015, sex-specific treatment practices changed, with increased use of EVR in women over time. Women who underwent EVR were more likely to be poor candidates for open repair (*p* < 0.001). Short-term (1 year) and long-term (10 year) survival rates were poorer in women after both open and endovascular repair, although sex-based differences were more significant after the latter. Although no significant sex differences were observed with open repair for elective or symptomatic aneurysms, women who underwent open repair for ruptured AAA had a greater risk of death, which was not statistically significant with endovascular intervention [75].

### 4.3. Lower Extremity Arterial Disease

Lower extremity arterial disease (LEAD), also called lower extremity peripheral artery disease (LEPAD), is the third component of the manifestations of systemic atherosclerosis [76]. Moreover, it is the main cause of lower limb amputation and a major risk factor for cardiovascular mortality [77,78]. Women with LEAD are on average 10–20 years older than men. It pertains to the diminished vascular benefits of estrogen, leading to a reduction in vasodilation and the absence of its antioxidant properties. Common risk factors for LEAD persist across sex, encompassing smoking, hypertension, diabetes mellitus, and dyslipidemia. However, in the case of women, the duration of smoking emerged as a risk factor for LEAD after just 10 years, in contrast to the 30-year threshold observed in men [79]. Despite that, other risk factors associated with LEAD in women have been shown to include obesity, osteopenia/osteoporosis, hypothyroidism, use of oral contraceptives, hormone replacement therapy, and complications associated with pregnancy (pre-eclampsia, gestational hypertension, placental abruption, and placental infarction) [80]. In addition, women with diabetes exhibit an increased hypercoagulable state, impaired endothelium-dependent vasodilation, more pronounced atherogenic dyslipidemia, and a higher prevalence of metabolic syndrome compared to diabetic men [81].

Recent epidemiological investigations indicate that women exhibit a prevalence of LEAD comparable to, if not higher than, that observed in men. This pattern is evident across diverse populations, encompassing women from low- and middle-income countries (LMIC) and those in socioeconomically disadvantaged groups. Nevertheless, it is crucial to acknowledge the potential underestimation of these figures, given that women frequently display asymptomatic or atypical symptoms in contrast to men, posing challenges in accurate diagnosis [82,83]. Symptoms in women, often resembling arthritis, neuropathy, or spinal stenosis, can be misconstrued as atypical manifestations of LEAD [84].

Furthermore, there is evidence indicating a higher prevalence of asymptomatic LEAD in women compared to men [80,81,84]. Moreover, women also have twice the prevalence of limb-threatening ischemia (CLTI) and more multilevel arterial occlusive disease [85,86]. Porras et al. [87], in their systematic review and meta-analysis, revealed that women diagnosed with LEAD tend to experience rest pain more frequently, with a lower prevalence of intermittent claudication.

Women have a greater functional impairment of the lower extremities, with a shorter distance from the treadmill to intermittent claudication, a shorter maximum distance from the treadmill, and a poorer quality of life compared to men [80,88]. The diminished treadmill claudication distances observed in women with LEAD may be attributed to their lower cardiopulmonary fitness and a self-perceived ability to climb stairs that is inferior to that of men. Consequently, Gardner et al. emphasize the importance of prioritizing exercise rehabilitation for women with intermittent claudication as a subgroup of LEAD patients, aiming to enhance their physical function [88]. Furthermore, the typical presentation of intermittent claudication in women generally occurs about 10–20 years later in age than men, and post-menopause [89].

In some cases, in women, making a diagnosis of LEAD can be a challenge. Women exhibit distinct fat distribution patterns in the lower body, limbs, and hips compared to men. This unique distribution can pose challenges for conducting precise physical examinations, including the assessment of pulses and bruits [90]. Additionally, duplex ultrasound may have some limitations in the examination of women. The high calcified arteries reported in women with diabetes and the elderly can cause difficulty in assessing the lumen of the artery. Moreover, in certain instances of obesity, the precision of this approach may be compromised, particularly in the proximal aorto-iliac arterial segments. Additionally, women demonstrate a higher susceptibility to adverse drug reactions associated with iodinated contrast media compared to men. This susceptibility may impose limitations on the utilization of CT and contrast angiography for the diagnosis of LEAD in women. In addition, some women having ABI > 1.4, as a consequence of arterial stiffness (patients with diabetes and end-stage renal disease, which occur more frequently in women) require alternative tests to diagnose LEAD [90].

Moreover, it was also reported that women with peripheral vascular diseases have low levels of knowledge and awareness about vascular diseases [91]. There are also differences between women and men concerning the use of guideline-directed medical therapy (GDMT)/optimal medical treatment. Women face a lower likelihood of being prescribed statins, antiplatelet agents, and angiotensin-converting enzyme inhibitors compared to men [92,93]. Even among high-risk patients with concurrent diabetes mellitus or chronic kidney disease, women are less likely to receive ACEi/ARB than men. Additionally, among individuals with diabetes, women exhibit inferior glycemic control compared to men. Furthermore, Black women are less frequently prescribed guideline-directed medical therapy (GDMT) compared to White women [94]. Additionally, fewer women undergo surgical intervention for LEAD [95]. They often have endovascular procedures. Nonetheless, women experience increased in-hospital complications following endovascular surgery, marked by elevated occurrences of bleeding, complications at the vessel access site, hematoma, or pseudoaneurysm [95]. In addition, women, after endovascular intervention, also have a higher risk of myocardial infarction, dissection, amputation, and death [96]. In the study by Hasan et al., it was shown that in women with LEAD who underwent peripheral vascular intervention (PVI), female gender was an independent predictor of major adverse cardiovascular events (MACE), mortality, non-fatal stroke, major bleeding, and higher cost. In turn, there were no significant differences in the rates of myocardial infarction, vascular complications, limb amputation, acute kidney injury, or length of stay [97]. However, Parvar et al. showed higher mortality and MACE rates in men with LEAD despite other accepted gender disparities [98].

A report from VASCUNET and the International Consortium of Vascular Registry presented data on symptomatic LEAD open surgical revascularization and peripheral vascular intervention (PVI) from 2010 to 2017. The information was gathered from administrative and registry data in the populations of 11 countries. Notable differences between women and men were evident in terms of patient age (72 vs. 70 years), the percentage of octogenarians (28% vs. 15%), the prevalence of claudication (45% vs. 51%), the incidence of PVI (57% vs. 51%), and the duration of hospital stay (7 days vs. 6 days) [99].

In another study, it has been shown that among claudication patients that women had a higher risk-adjusted rate of major amputation but a lower risk of mortality. However, among CLTI patients, women had a lower risk-adjusted hazard of major amputation [100].

### 4.4. Vasculitis

Vasculitis is a heterogenous group of diseases defined by the presence of leucocytic inflammatory infiltrate in the vessel walls with reactive damage to the mural structures and necrosis. The endothelial injury can lead to thrombosis and ischemic tissue damage while the damage to endothelial wall can cause aneurysm and perforation with subsequent hemorrhage [101]. The most frequently observed vasculitis in women is Takayasu Arteritis (TAK), also known as ‘pulseless women’ disease, which is a systemic inflammatory condition leading to damage to the medium and large arteries and their branches. It is a rare disease with a reported worldwide incidence rate of only 1 to 2 per million. It occurs predominantly in young Asian girls and women under 40 years of age. Female preponderance is characteristic of TAK (9:1). TAK exhibits a panarteritis pattern, initiating around the vasa vasorum and the medioadventitial junction. Early stages show active inflammation with mononuclear cell infiltration, elastic fiber fragmentation, and giant cell granulomatous reaction. Later phases involve reactive fibrosis, increased ground substance, mural thrombus, and neovascularization. Severe inflammation can lead to smooth muscle cell loss, medial weakening, vascular dilatation, and aneurysm formation. In the healed phase, adventitial fibrosis, scarring, and laminar medial necrosis become prominent, surpassing those seen in other aortic inflammatory disorders. Clinical manifestations of TAK vary with disease stage and vascular region involvement. The disease progresses through three stages: Stage I, the “prepulseless” phase, marked by constitutional symptoms; Stage II, the “pulseless” phase, characterized by vascular inflammation symptoms; and Stage III, the “fibrotic” stage, involving complications from vascular damage. Few patients reach the third stage, and only 19% follow a triphasic progression through all three stages [102]. Specific symptoms depend on the affected vessel and are detailed in Table 1.

The 2018 EULAR guidelines for the pharmacological treatment of TAK recommend a two-phase strategy, with a combination of conventional glucocorticoid and synthetic DMARD in phase I, and a biologic DMARD in phase II if relapse occurs [103]. The 2021 American College of Rheumatology guidelines also recommend glucocorticoid combination therapy with DMARDs but differ in recommending tumor necrosis factor inhibitors as an option in initial therapy; tocilizumab is recommended in refractory disease [104]. Antiplatelet therapy is associated with a lower frequency of ischemic events but with an increase in bleeding. It is recommended in critical cerebrovascular disease or in vertebrobasilar involvement, but not routinely [104]. Vascular lesions in TAK are responsible for most of the morbidity and mortality associated with the disease, but current guidelines favor restricting invasive therapy (open surgery or endovascular treatment) in TAK to life- or organ-threatening situations, refractory hypertension, or when patient activities are significantly affected [102].

### 4.5. Vasospastic Diseases

Vascular dysregulation, the so-called vasospastic syndrome (VSS), occurs in the context of subjects who have a tendency to respond to stimuli such as coldness or emotional stress with inappropriate vasoconstriction or insufficient vasodilation in the microcirculation. Individuals experiencing vasospastic syndrome typically exhibit cold hands and feet along with abnormal vasoconstriction following exposure to local cold. Women are more commonly affected than men, and the initial symptoms often manifest during puberty, diminishing with age, particularly after menopause. The syndrome appears to have a hereditary component. Key symptoms include cold hands and, occasionally, cold feet. Elevated plasma ET-1 levels have been noted in those with vascular dysregulation [105]. Diagnosis methods for vasospastic syndrome include nailfold capillaroscopy combined with cold provocation. Vasospastic diseases like Raynaud’s syndrome, acrocyanosis, and erythromelalgia vary in prevalence, clinical presentation, therapy, prognosis, and impact on quality of life. **Raynaud’s syndrome**, affecting 5 to 20% of the European population, is more common in women, typically emerging around the age of 40. Attacks involve transient white-blue-red or white and blue discoloration of fingers and toes, triggered by cold or stress, lasting minutes to hours. Primary, secondary (with known causes), and suspected secondary Raynaud’s syndromes are differentiated. Treatment options include vasodilators, particularly long-acting calcium channel blockers. **Acrocyanosis**, a vasospastic acral disorder, results in persistent reddish-livid discoloration, primarily in hands and feet, with a higher incidence in women before the age of 25. Primary acrocyanosis lacks an identifiable cause, whereas secondary forms may respond to vasodilators and long-acting calcium channel blockers. Primary and secondary **erythromelalgia**, a rare condition, involves paroxysmal burning pain and redness in the legs, feet, and occasionally hands, triggered by warmth. Onset typically occurs between 40 and 55 years, with various therapeutic approaches that are occasionally successful [106].

### 4.6. Chronic Venous Insufficiency

Chronic venous insufficiency (CVI) is a vein disorder affecting millions of people every year. Women are more commonly affected than men [107]. Long-term inadequate venous function in the lower limbs presents a diverse range of clinical manifestations, spanning from asymptomatic cosmetic concerns to severe symptoms [108,109,110]. These can include telangiectases, reticular veins, varicose veins, edema, pigmentation and/or eczema, lipodermatosclerosis, atrophie blanche, and venous ulceration. Irregular venous flows in the lower extremities are detected in about 50% of individuals, with the estimated prevalence of Chronic Venous Insufficiency (CVI) varying depending on population studies [110].

Both men and women share common risk factors, including age, deep vein thrombosis, obesity, smoking, cancer, occupation, muscle weakness, leg injury, inactivity, family history, phlebitis, and footwear choices [111]. However, women, particularly during pregnancy and due to poor biomechanics related to footwear, are more susceptible to chronic venous insufficiency compared to men.

The main pathophysiological cause of the clinical manifestation of CVI of the lower extremities is ambulatory venous hypertension, which is caused by venous valve reflux, venous flow obstruction, or both [108,110,112].

To understand the pathophysiology of CVI or varicose veins, as well as their therapeutic options, such as endovenous ablations, one should know the anatomy and variations of the veins [97,98]. The venous system can be divided into three major components: the superficial venous system, the deep venous system, and perforating veins. The superficial veins are most frequently affected in patients with chronic venous disease [110,112].

Clinical manifestations of CVI encompass discomfort, swelling, varicose veins, and skin alterations or ulceration. Venous leg discomfort is commonly characterized as a dull ache, throbbing, heaviness, or pressure sensation, especially after prolonged standing, and can be alleviated by measures that reduce venous pressure, such as leg elevation, compression stockings, or walking. However, approximately 20% of patients with other CVI clinical features do not experience leg discomfort, while around 10% solely present with this symptom [93]. In individuals with varicose veins, tenderness may arise due to venous distension. Cutaneous changes involve skin hyperpigmentation, stasis dermatitis, and ulceration. Hemosiderin deposition causes hyperpigmentation. Notably, hyperpigmentation in nonvenous conditions, like acanthosis nigricans or hemosiderosis, tends to be more diffuse or affects other body areas. Lipodermatosclerosis denotes inflammation of subcutaneous fat. Distinguishing a venous ulcer from an ischemic ulcer involves noting that ischemic ulcers are typically deeper, often exhibiting gangrenous edges or a gangrenous base [110,112].

Establishing an accurate diagnosis of CVI relies on a thorough history and physical examination. The physical examination should be conducted in an upright position to maximize vein distension. Both non-invasive and invasive diagnostic tests play crucial roles in supporting the diagnosis. In patients with CVI, the DUS examination should reveal both the anatomical patterns of veins and abnormalities in venous blood flow within the limbs.

Conservative management is the initial approach for all patients displaying signs and/or symptoms of CVI. The primary component of conservative management is the use of compression stockings. Additionally, encouraging risk modifications, such as weight reduction in obese individuals, regular walking exercise, and smoking cessation, is essential as part of conservative treatment [110,112].

The use of medical treatment for CVD has been a practice for many years, although there is some debate about its precise role in the treatment approach. Venoactive drugs (VADs) find extensive prescriptions in certain countries but are not universally available. These drugs fall into two categories: natural and synthetic. The primary mechanisms of action for VADs include reducing capillary permeability, minimizing the release of inflammatory mediators, or enhancing venous tone [113]. Numerous compounds have undergone testing with varying degrees of success, but among the most promising drugs are Ruscus extracts, micronized purified flavonoid fraction, calcium dobesilate, horse chestnut extract, hydroxyethylrutosides, red vine leaf extract, and sulodexide [110,112].

Open surgical therapy of varicose veins with high ligation and stripping of the GSV combined with the excision of large varicose veins has been the standard of care for more than a century. During the past decade, endovenous ablation therapy has largely replaced this classic ligation and stripping.

There are two types of thermal ablation therapy: EVLA and RFA. Both are guided by ultrasound. The mechanism involves a heat generator that causes local thermal injury to the vein wall leading to thrombosis and fibrosis. EVLA and RFA showed the same safety and efficacy in terms of quality of life, occlusion, thrombophlebitis, hematoma, and recanalization after 1 year [110,112,113]. The most common complication is bruising, which is observed in up to 75% of patients who receive ablation therapy. Other potential but rare complications include superficial vein thrombosis, DVT (especially EHIT), skin burns, pigmentation, and nerve injury.

Sclerotherapy is the least invasive percutaneous technique, using chemical irritants to close unwanted veins. Telangiectases, reticular veins, small varicose veins, and venous segments with reflux can be treated with sclerotherapy [110,112].

Women must be mindful of chronic venous insufficiency, as lower extremity venous disease is a relatively common yet frequently overlooked medical issue. Given its association with a broad clinical spectrum, it is essential to approach patients with suspicion of this condition. A comprehensive understanding of the normal anatomy and functioning of the venous system is necessary to accurately comprehend and diagnose the pathophysiology of CVI. Functional assessment through Doppler ultrasound (DUS) is indispensable for diagnosing patients with CVI. Although compression stockings serve as a cornerstone in conservative management, low compliance poses a significant challenge to this therapy. Considering the symptomatic nature, earlier adoption of venous ablation therapy should be contemplated for these patients.

### 4.7. Venous Thromboembolism

The most important acute venous pathology is venous thromboembolism (VTE), which is the combined name for deep vein thrombosis (DVT) and pulmonary embolism (PE). VTE is the third most common cardiovascular disease, with an annual incidence of 100–200 per 100,000 people. The reported annual incidence rates of PE ± MVT and MVT-only cases vary from 29–78 and 45–117 per 100,000 population, respectively, according to different surveys. The prevalence of VTE increases with age, with a higher percentage in women at younger ages and in men at older ages. In women of reproductive age, VTE is a specific risk.

VTE is a major cause of morbidity and mortality during pregnancy and the postpartum period. The relative risk of VTE increases 5-fold during pregnancy and 60-fold in the puerperium (6 weeks postpartum) [114]. Incidence of VTE increases slightly above that of the general population in the first trimester, rises to a greater degree during the third trimester, and peaks in the first two weeks after delivery. Approximately half of all pregnancy-associated VTEs occur postpartum. Pregnancy-associated risk factors for VTE include cesarean delivery, assisted reproductive technology, stillbirth, preterm birth, preeclampsia, obstetric hemorrhage, and postpartum infection. Medical conditions associated with antepartum or postpartum VTE include preexisting diabetes mellitus, inflammatory bowel disease, systemic lupus erythematosus, and sickle cell disease. More than half of pregnancy-related VTEs are associated with an underlying thrombophilia [115].

In women, hormonal contraception also significantly increases the risk of VTE, especially when combined with other risk factors such as obesity, smoking, or hereditary thrombophilia. Increased risk of thrombosis is also associated with ovarian stimulation therapy, in the background of which studies have demonstrated the effect of supraphysiological estrogen levels on various coagulation factors [116]. Postmenopausal hormone replacement therapy is associated with a 2–4-fold increased risk of VTE [117]. Patient-associated predisposing risk factors and acute triggers for VTE are listed in Table 2.

Once the acute trigger has disappeared, the associated increased risk of thrombosis disappears, whereas persistent predisposing risk factors play an important role in the recurrence of VTE.

VTE recurs in about 30% of patients in the 10 years following an acute event, regardless of the length of acute treatment. Independent predictors of recurrence are age, male sex, active malignancy, and neurological disease with lower limb paralysis. Other predictors of recurrence include idiopathic VTE, lupus anticoagulant or antiphospholipid antibody, antithrombin, protein C or protein S deficiency, hyperhomocysteinemia, persistently elevated plasma D-dimer in idiopathic VTE, and residual venous thrombosis [118]. Various VTE recurrence-prediction scores have been developed to assess the risk of recurrence, particularly in patients with idiopathic or cancer-related VTE. In women with idiopathic VTE, if up to one of the following risk factors was identified, the risk of recurrence of VTE was significantly lower: (1) older age (≥65 years), (2) obesity (BMI ≥ 30 kg/m^2^), (3) increased D-dimer before stopping warfarin therapy, and (4) signs of post-thrombotic syndrome. The risk of recurrence after discontinuation of anticoagulation for a combined oral contraceptive-associated VTE was low, and this was lower than after an unprovoked VTE [119]. Persistently elevated D-dimer after discontinuation of anticoagulant therapy, age group over 50 years, male sex, and VTE unrelated to hormonal therapy (in women) increased the risk of recurrence of idiopathic VTE in the DASH prediction score [120].

Typical symptoms of lower limb DVT include swelling, pain, redness, and dilatation of the superficial veins in the affected limb, although sometimes the disease may be asymptomatic. Scoring systems (most commonly the Wells score) used to determine the probability of DVT play an important role in its diagnosis, with DVT being classified as high, medium, or low probability. The “gold standard” for objective confirmation of DVT is the compression ultrasound (CUS). Pregnant patients with normal proximal CUS results should have dedicated iliac vein testing (typically Doppler/duplex ultrasonography or, rarely, non-contrast MRI) if they have symptoms of iliac vein DVT [115]. For the evaluation of PE, computed tomography pulmonary angiography (CTPA) has become the diagnostic-imaging standard. Planar lung ventilation/perfusion scintigraphy is a common test in pregnancy. Most young pregnant women do not have significant lung disease, and guidelines support the use of perfusion-only scintigraphy (i.e., no ventilation scan) in pregnant patients with normal chest radiographs to reduce maternal and fetal radiation exposure [115]. Among the laboratory tests, an elevated (>0.5 mg/mL) quantitative D-dimer level supports but does not confirm the diagnosis of VTE, as it can be elevated in many other conditions (postoperative, infectious, liver disease, heart failure, cancer, pregnancy, and DIC).

Treatment of DVT is based on anticoagulation. The agent used in the acute phase may be subcutaneous LMWH, intravenous or subcutaneous unfractionated heparin (UFH), or subcutaneous fondaparinux. In parallel to being given oral vitamin K antagonist (VKA) treatment, parenteral anticoagulation should be continued until the INR is between 2 and 3 on two consecutive days, but for at least 5 days. There are also new or direct (DOAC) oral anticoagulants available for the treatment of VTE, which act by inhibiting fibrin (dabigatran) or activated factor X (apixaban, edoxaban, and rivaroxaban). They have the advantage of not requiring INR control, having no food interactions, and having significantly reduced drug interactions. The duration of anticoagulant treatment is known, with 3 months for transient cause, at least 6 months for unknown cause, and at least 12 months for recurrent DVT. The duration of treatment is also influenced by the degree of recanalization detectable by duplex ultrasound examination. Most cases of pregnancy-related VTE can be treated with low molecular weight heparin, but cases of limb- or life-threatening VTE require consideration of thrombolysis and other reperfusion therapies [121].

Obstetricians–gynecologists, infertility, and menopausal specialists have key roles in the prevention of VTE in women, and in taking into account the increased risk of VTE in treated women.

### 4.8. Lymphedema

Lymphedema is a prevalent (2–4/1000) [122] but frequently overlooked disease caused by an imbalance between the production and reabsorption of lymphatic fluid due to disruption or overload of the lymphatic system. It can be a primary manifestation of congenitally abnormal lymphatic vessels or can be secondary to other conditions that damage the lymphatic vessels or lymph nodes. Although multiple factors intervene in its pathophysiology, the lymphatic endothelium expresses estrogen receptors, suggesting a role of estrogen in the lymphatic function that may explain the higher prevalence in females (60–80% of the population) [122,123,124,125].

Individuals born with agenesis or hypogenesis of the lymphatic vessels will develop lymphedema at a different age, either alone or within a syndrome (Noonan, Turner, etc.). Primary lymphedema is rare (1–10%) and more prevalent in females after puberty, suggesting hormonal influence [123,126]. Penetrance is variable, and in some patients it may develop after a triggering event [127].

Worldwide, it is caused most by filarial infection. In developed countries, common causes of secondary lymphedema include cancer and cancer therapies, followed by obesity, infection, and inflammatory disorders. It can appear years after the initiating event. Cancer-associated lymphedema is more common in females: prevalence is high in breast cancer (13–20%) and gynecological malignancies (20–37%) compared to other malignancies equally distributed by gender [128,129]. The risk of lymphedema is influenced by the therapy, being higher for lymph node resection and radiation. Tamoxifen, a partial estrogen receptor agonist used in hormone-dependent breast cancer has been associated with lymphatic dysfunction and may predispose to lymphedema [126].

Edema affecting one or both limbs is the main clinical finding. Patients describe tightness, heaviness, or discomfort. Initially, the edema is soft, pitting, and improves with elevation. As lymphedema progresses, it becomes constant and non-pitting due to cutaneous fibrosis and adipose deposition. The skin thickens, as evidenced by Stemmer’s sign (the inability to pinch the skin below the second toe) and develops hyperkeratosis with verrucose lesions. In females with breast cancer-associated lymphedema, more than 60% reported that it affected their body imaging and sexuality [130]. A common theme in patient forums revolves around finding suitable options for clothing to accommodate for the size discrepancy of the limbs and compression garments.

Lipedema, almost exclusively affecting females, can be mistaken for lymphedema; however, both conditions may overlap. Lipedema impacts quality of life, social, and emotional functioning. Its diagnosis is frequently delayed, as is the appropriate management and support [131]. The presence of port-wine stains or limb discrepancy should raise concern for Klippel–Trenaunay–Weber syndrome, vascular malformations, or hemihypertrophy.

Diagnosis can be delayed, as often body changes are attributed to obesity. Typical clinical features in the right clinical setting establish the diagnosis of lymphedema. Surveillance of patients at risk, (breast cancer, gynecological malignancies) may help to reduce time lapses in diagnosis and treatment.

Objective measurements of the volume of the affected limb obtained with water displacement methods, circumferential measurements, or electronic volumetry are helpful for assessing the effectiveness of therapies [129]. Lymphoscintigraphy is commonly used to assess the lymphatic flow. It obtains serial images after administration of a radiolabeled agent, distally, in the affected limb.

Complex decongestive therapy is the cornerstone of lymphedema therapy regardless of the underlying reason, and its goal is to achieve and maintain maximal volume reduction in the affected limb. This multi-modality therapy is performed by a specialized therapist in multiple sessions and it includes manual lymphatic drainage, multilayer bandaging, decongestive exercises, and chronic use of compression garments. Pneumatic pumps providing intermittent compression specially designed for lymphedema therapy can be used for maintenance, in addition to compression garments. These treatment modalities are time consuming and require lifelong commitment.

Surgical therapy can be considered in selected patients, directed at restoring lymphatic flow in the early stages of the disease [129] or at reducing the limb size, in severe forms of lymphedema, in addition to compression garments [132].

Most studies focusing on quality of life and psychosocial consequences of lymphedema have been conducted in women with lymphedema after breast cancer and its treatment. Among breast cancer survivors, patients with lymphedema had significantly higher medical costs, hospitalizations, and medical visits than those without lymphedema [133,134]. Among female survivors of other cancers, those with lymphedema reported lower physical function and required more assistance for activities of daily living [135]. Lifting restrictions, limited mobility, and fear of infections restrict the possibilities of actively participating in work, hobbies, or sports. Other recurring themes that describe the impacts of lymphedema on daily life include altered body image, sleep disturbances (avoiding the affected site, elevation of the limb during sleep), burden of self-care (economic cost, time-consuming tasks, and need to alter their wardrobe), and the impact of living with a chronic disease [136,137].

In addition to this, patients with lymphedema are predisposed to having recurrent cellulitis: 38% had at least one episode and 23% had more than one [138]. Infections lead to the progression of symptoms through lymphatic damage. Treatment of edema, prevention of injuries, and treatment of tinea pedis are recommended to decrease the risk of cellulitis [138,139,140]. Rarely, women treated for breast cancer with radiation therapy can develop a rare and aggressive angiosarcoma (Stewart-Treves syndrome). Skin changes should prompt a further evaluation [141].

In conclusion, lymphedema disproportionately affects women and their physical, psychosocial, and emotional well-being. It is a chronic physical and economic burden for these patients, which deserves research and resources for prevention, understanding the challenges, and exploring innovative therapies.

### 4.9. Pelvic Congestion Syndrome

Pelvic congestion syndrome (PCS) is a venous disorder characterized by chronic non-cyclic pelvic pain caused by venous insufficiency predominantly associated with pelvic varicosities in women [142]. Environmental factors such as pregnancy, obesity, jobs associated with prolonged standing or heavy lifting, some treatment interventions such as pelvic surgery and estrogen therapy, some anomalies in pelvic venous anatomy, and a genetic basis are risk factors involved in the pathology of PCS [143,144,145].

There is a spectrum of clinical manifestations in PCS patients that can make PCS diagnosis challenging. This spectrum of clinical manifestations presents as various combinations of anatomical zones, etiology, and hemodynamic disturbances. Therefore, to avoid misdiagnosis and missing rare cases, we need to consider patient symptoms based on the main elements of PCS, including anatomical zones, etiology, and hemodynamic disturbances [144,146].

There are four anatomical zones in the abdomen and pelvis that are related to the pathophysiology and consequently symptoms of PCS, including the left renal vein; the gonadal, internal iliac and pelvic veins; the pelvic origin extra-pelvic veins; and the lower extremity veins. The pathophysiology of PCS is considered in three domains of the anatomic, hemodynamic, and etiologic. Based on the SVP classification, which includes symptoms (S), varices (V), and a pathophysiologic domain, which is a composite anatomic-hemodynamic-etiologic domain (P), the inferior vena cava, left renal vein, gonadal veins, iliac veins, and pelvic escape veins are anatomic segments of this classification. Also, the underlying pathological hemodynamic can be reflux or obstruction, and the etiology can be thrombotic, non-thrombotic, or congenital [144,146]. In addition, there are other factors involved in the pathogenesis of PCS, including estrogen, inflammation, vasoactive peptides such as endothelin, calcitonin gene-related peptide (CGRP) and substance P (SP), and nociceptive mechanism, which activates the autonomic nervous system by sexual and non-sexual stimuli, and neuropathic and psychogenic mechanisms [147,148].

According to anatomical zones, there are three categories of symptoms, including venous renal symptoms, chronic pelvic pain, and extra-pelvic pain. Left renal vein compression (usually associated with nutcracker syndrome) can lead to renal venous hypertension, microhematuria or macrohematuria, and left flank or abdominal pain that is worsened by activities such as standing, sitting, or walking. Symptoms of this category are more prevalent on the left side. The second category of symptoms are characterized by chronic pelvic pain characterized as a dull unilateral or bilateral pain associated with negative cognitive, behavioral, sexual, and emotional consequences and symptoms related to lower urinary tract, sexual, bowel, pelvic floor, myofascial, or gynecologic dysfunction. Symptoms are often worse with activities such as walking and prolonged standing and improve with lying down. Symptoms of this category are sensitive but non-specific. The third group of symptoms is subdivided into symptoms localized to the external genitalia or lower extremities, including reflux-related symptoms, symptoms related to posteromedial thigh, sciatic/tibial nerve varices, and venous claudication associated with iliocaval venous obstruction. Also, renal hilar varices, pelvic varices, and pelvic origin extra-pelvic varices are varices classifications related to the anatomical zones [142,144,146].

In addition to considering the clinical manifestations as a spectrum and performing SVP classification, the first line of diagnosis includes non-invasive approaches, including pelvic ultrasound, cross-sectional computed tomography (CT) and magnetic resonance (MR) imaging, whereas conventional venography is the gold standard procedure for PCS diagnosis [142,149]. Depending on the SVP class, which is diagnosed and confirmed by imaging, there are different options for treatment, including pharmacological and hormonal therapy, endovascular therapy, and surgery [149,150].

PCS diagnosis is common in young women of reproductive age. Chronic pain and reduced physical activities are the main factors that affect the quality of life of PCS patients by limiting their social activity and the possibility of regular work. Furthermore, there is no explanation for the chronic pelvic pain of about 61% of patients. The fact that these patients do not know the reason for their pain makes their life harder than the group that is aware of their diagnosis [147,151].

In addition to social life, they have major challenges in family life with sexual challenges, infertility, and pregnancy. Symptoms such as dysmenorrhea, dysuria, and dyspareunia can affect sexual function [151]. In addition, some case reports indicate that persistent genital arousal can be associated with PCS [152,153]. Infertility is another challenge for PCS patients due to ovarian varices or pelvic congestion caused by inferior vena cava obstruction [142,154,155]. As mentioned below, pregnancy is a risk factor for PCS. Compression of the iliac veins by the gravid uterus, elevation of blood volume, and dilation of the ovarian and pelvic veins during pregnancy are the factors that make multiparous women prone to PCS [142,145,156]. In addition, the pain that worsens during pregnancy makes pregnancy difficult for PCS patients [157]. There is a lack of knowledge about the risk of deep vein thrombosis during pregnancy in PCS patients. These limitations and challenges in different aspects of the life of the PCS patient can affect their quality of life and induce psychological pressures that can not only affect their mental health, but also affect the circulation of the pelvis due to vasodilation [148,151].

Although PCS is well known as a premenopausal disease in which there is a remission of symptoms, possibly due to less estrogen and consequently a decrease in vein dilation, there are postmenopausal patients who meet the PCS criteria [156,157,158].

Taken together, the spectrum nature of PCS indicates the necessity of considering all of the anatomical, hemodynamical, and etiological combinations in order to avoid misdiagnosis and missing rare cases. Furthermore, interpreting a PCS patient’s condition needs a holistic view considering different aspects including neurochemical mechanisms; anatomical anomalies; psychological features; patient’s personality, job and desires; sexual function and response to sexual and non-sexual emotions; and change in position.

### 4.10. Fibromuscular Dysplasia

According to the definition of the European consensus, fibromuscular dysplasia (FMD) is ‘idiopathic, segmental, non-atherosclerotic and non-inflammatory disease of the musculature of the arterial walls, leading to stenosis of small and medium arteries’ [159,160].

FMD most commonly affects the cerebrovascular and renal arteries, but most arteries may be involved throughout the body [160].

Histological findings show fibrosis, cellular hyperplasia, and distorted architecture of the arterial wall. Due to a relative paucity of inflammatory cells in advanced lesions, FMD is defined as a ‘non-inflammatory’ disease, but inflammation could be involved in the early stages of the disease [161].

**Figure 1:**
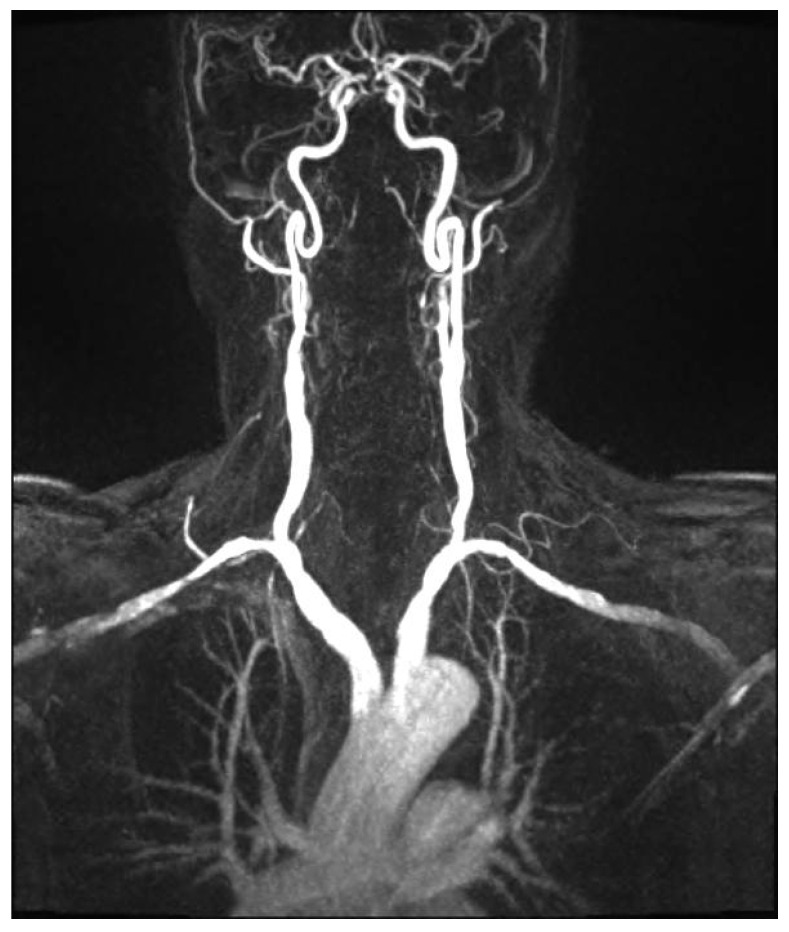
Tortuosity of the ICAs and S-curve (MRA).

**Figure 2:**
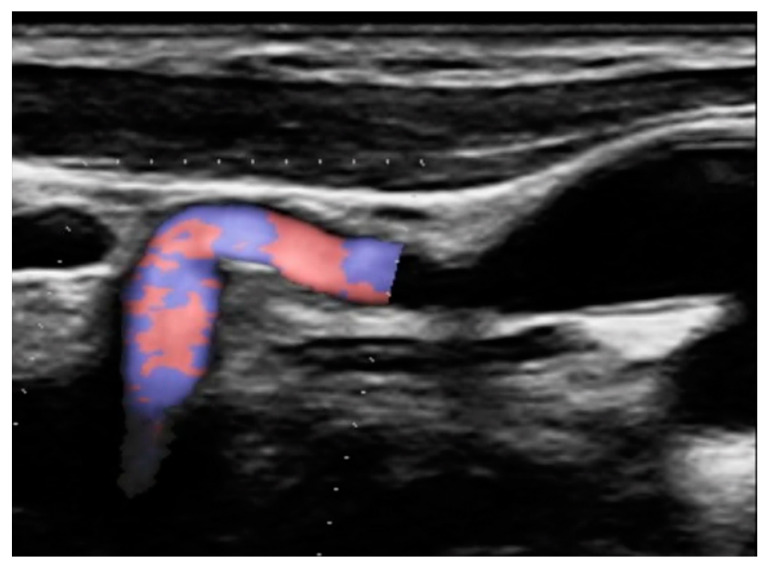
Angulation of the post-bulbar ICA (DUS).

Since the advent of endovascular procedures, the histopathological classification has been replaced by an angiographic classification: focal or multifocal FMD. In multifocal FMD, areas of stenosis alternate with areas of dilation (“string of beads”). They are usually located in the mid and distal portions of the vessel [160,161]. Recently, aneurysm, dissection, and arterial tortuosity have been included in the phenotype of FMD. However, a diagnosis of FMD cannot be established in the absence of focal or multifocal stenosis. Tortuosities can also be seen in the carotid, vertebral, and renal arteries. Sinuosity of the mid to distal portion of the internal carotid artery (ICA) may lead to an “S-curve”. It is not specific for FMD, but it should alert the clinician in individuals <70 years of age [162]. Figure 1 and Figure 2.

#### 4.10.1. Epidemiology, Pathogenesis, and Genetics

Although approximately 82–95% of patients with FMD are women, men can also develop FMD with a higher frequency of aneurysms and dissection, especially if they are current smokers. FMD can occur at any age, but the mean age at diagnosis is 43–53 years [161,163]. Notably, 3–6% of the patients are asymptomatic.

There are sporadic and familial forms, but only 1.9–7.3% of patients report an affected family member. The pathogenesis of FMD is poorly understood. FMD is probably linked to an association of environmental and genetic factors. Tobacco smoking may also be a potential pathogenic factor, but the data is still equivocal. The use of fluoroquinolones weakens the vessel wall and increases the risk of thoracic and abdominal aneurysms, and spontaneous cervical artery dissection. While waiting for specific data in FMD patients, and according to the FDA, “fluoroquinolones should not be used in patients at increased risk of aortic ruptures or dissections unless there are no other treatment options available” [161].

Recently, a single nucleotide polymorphism, rs9349379-A, in the Phosphatase and Actin Regulator 1 (*PHACTR1*) gene has been identified as a common genetic risk variant. This risk locus is associated with spontaneous coronary dissection (SCAD), carotid artery dissection, hypertension, and migraine headache. Interestingly, FMD, migraine, SCAD, and related diseases are also more frequent in women. On the contrary, the risk locus has an inverse association with coronary atherosclerotic arterial disease, and patients with FMD appear to have fewer carotid plaques. Patients with FMD might be protected from atherosclerosis. Other rare genetic variants have also been implicated in FMD, but further genetic studies are needed. Currently, there is no specific genetic test for FMD. Genetic testing of asymptomatic relatives of patients is not advised, but relatives should undergo clinical exam [160,161].

#### 4.10.2. Renal FMD

Its prevalence is estimated between 3 and 6%. Renal arteries are involved in about 75% of FMD patients. The typical phenotype is “middle-aged white woman with personal and family history of hypertension”. Up to 90% of these women have multifocal FMD. Focal FMD is usually diagnosed in people under 30 years of age, with higher blood pressure and a more balanced sex distribution than multifocal FMD patients [160]. Unlike atherosclerotic renal artery stenosis (ARAS), in patients with “string of beads” lesions, renal function and intrarenal microvascular function are usually preserved, even in the case of unilateral tight stenosis. Conversely, the impact of focal FMD is more severe but the success rate of balloon angioplasty is higher. (161) With a better spatial resolution and a better visualization of calcifications than magnetic resonance angiography (MRA), computed tomographic angiography (CTA) is the first-line imaging technique. MRA is an alternative when CTA is contraindicated. Duplex ultrasound (DUS) requires extensive expertise. Catheter-based angiography may be helpful when the severity of the stenosis is difficult to assess (especially for multifocal lesions) or as a control after angioplasty. It should be combined with translesional pressure-gradient measurement. Some experienced centers can combine angiography with intravascular ultrasound (IVUS) or optical coherence tomography (OCT) [160].

#### 4.10.3. Cerebrovascular FMD

**Figure 3:**
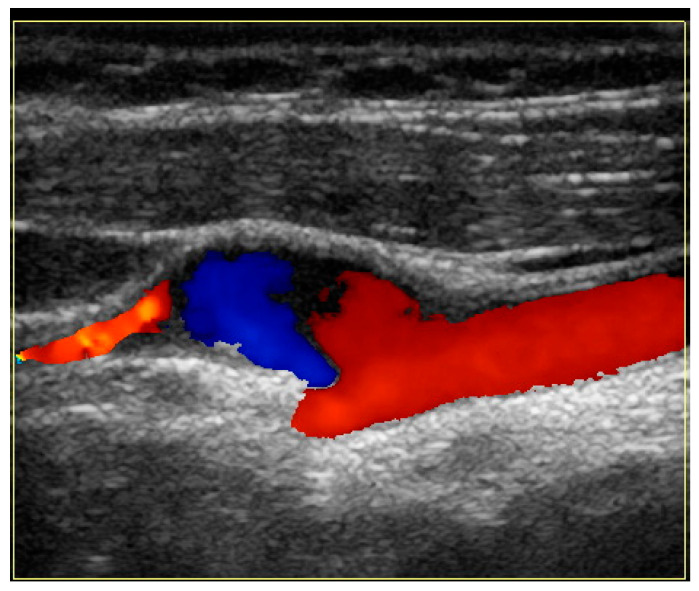
Dissection of the post-bulbar ICA (DUS).

Headaches are the most frequent but non-specific symptoms (50–70% of patients with FMD) [160,164]. Pulsatile tinnitus may be associated with cervical artery dissection. The prevalence of cervical artery dissection and intracranial saccular aneurysm is higher among patients with FMD, with a higher rate of neurological complications (TIA, ischemic stroke, subarachnoid hemorrhage) (Figure 3).

**Figure 4:**
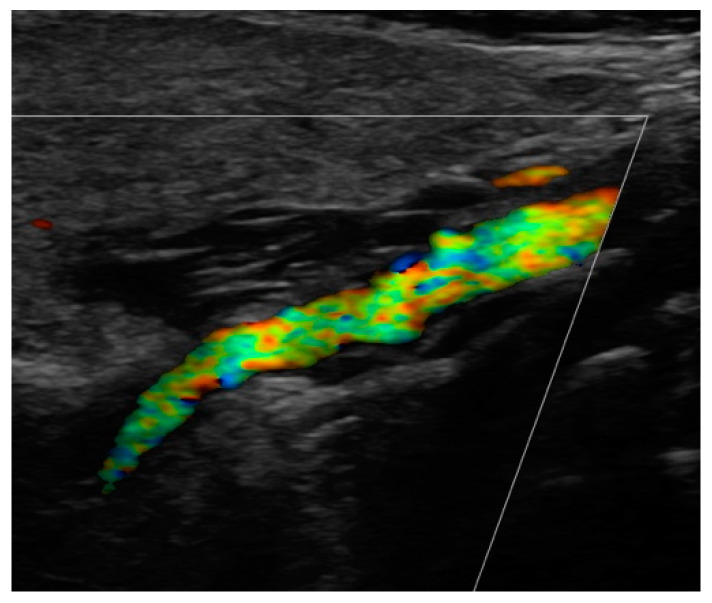
Multifocal FMD of the post-bulbar ICA (DUS).

**Figure 5:**
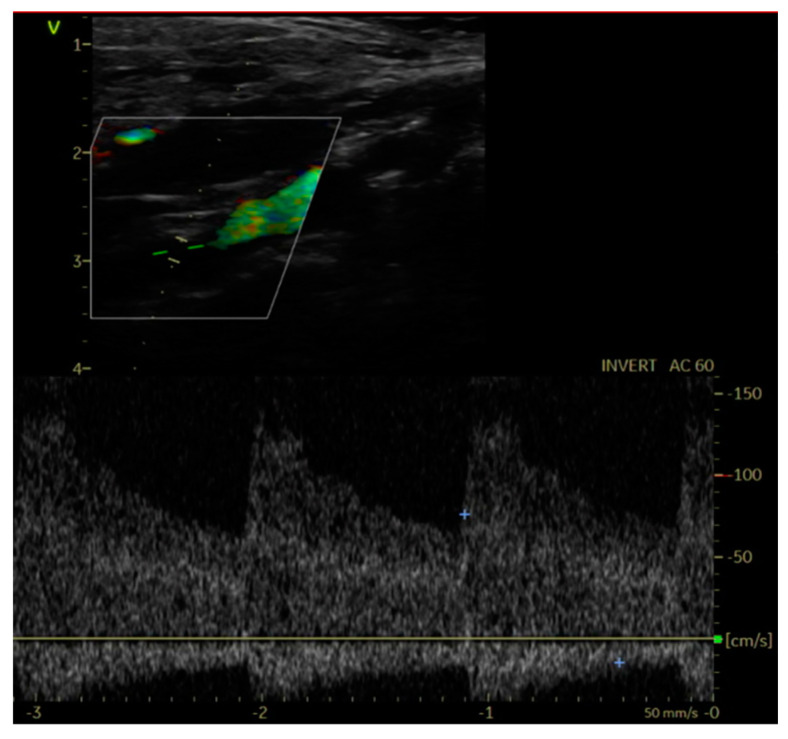
High−velocity flow in FMD stenosis (PW Doppler).

The risk of long-term progression of FMD and the occurrence of aneurysm and dissection are not well known [164]. CTA and MRA are the first-line imaging modalities. Catheter-based angiography must be reserved for patients that may require intervention. Carotid DUS maybe useful for surveillance, but with some drawbacks: unsatisfactory access to vertebral and carotid arteries (especially the distal cervical portion of the ICA and intracranial arteries), and no validated criteria for FMD [160] (Figure 4 and Figure 5).

#### 4.10.4. Spontaneous Coronary Dissection (SCAD) and FMD

SCAD is an important cause of “myocardial infarction with nonobstructive coronary artery disease”, called MINOCA. Most of these patients are young to middle-aged women, and almost half of them suffer from FMD. However, although SCAD and FMD show overlapping data, they are not a single disease [165].

#### 4.10.5. Pregnancy-Related Complications

In the FEIRI Registry, the high prevalence of gestational hypertension (25%) and preterm birth (20%) was probably related to renal FMD. However, the prevalences of preeclampsia and arterial complications were low to moderate in this population [166].

### 4.11. Diabetic Angiopathy

The global burden of diabetes mellitus has increased significantly over the last several decades. The increase in diabetes incidence rate is projected to continue parallel to the increasing obesity epidemic. Although young and middle-aged men show a higher prevalence of Type 2 diabetes mellitus than their counterpart women, postprandial hyperglycemia increases to a larger extent in women as they age. Consequently, women show a higher prevalence of undiagnosed diabetes after 60 years of age and of total diabetes after 70 years of age [167].

A major complication of diabetes is blood vessel disease, termed angiopathy. Patients with diabetes mellitus are at increased risk of both adverse cardiovascular and microvascular complications. Each macro- or microvascular disorder can be considered to be a tissue-specific manifestation of the glucose-driven pathogenetic processes occurring at susceptible sites in the body. Macrovascular diseases comprise coronary heart disease (CHD), peripheral arterial disease (PAD) and stroke. Microvascular complications of diabetes include diabetic retinopathy, diabetic nephropathy, and diabetic neuropathy. Recent evidence suggest sex differences in the prevalence, progression, and pathophysiology of diabetes-driven macrovascular and microvascular diseases. The consequences of macrovascular complications may be greater in women. Although scarce and inconclusive, some evidence suggest that men might be at a higher risk for diabetic microvascular complications [168].

Diabetes is among the strongest risk factors for risk of future CHD. In the INTERHEART global case–control study of >27,000 individuals, diabetes mellitus was associated with a higher risk of myocardial infarction (MI) in women than any other risk factor. The association of diabetes mellitus with the risk of stroke is complex due to the variety of stroke types. Among 116,316 women from the Nurses’ Health Study, aged 30 to 55 years and followed for 26 years, Type 1 diabetes mellitus was associated with a greater risk of ischemic and hemorrhagic strokes, while Type 2 diabetes mellitus was associated with a greater risk of ischemic stroke but not hemorrhagic stroke [169]. Recent evidence indicates that the risk of stroke is increased in diabetic patients with hyperglycemia but not in those without hyperglycemia, suggesting an important role for metabolic disturbances in these associations [170]. Diabetes mellitus also deteriorates the outcomes of stroke and coronary heart disease. Similarly to other manifestations of atherosclerosis, diabetes is strongly linked to PAD, increasing the risk of PAD by 2 to 4 times. Unlike other forms of cardiovascular disease, diabetes does not appear to confer a higher excess risk in women compared to men for PAD [171].

Men show a faster progression of diabetic nephropathy and are more often treated with dialysis. However, during chronic dialysis treatment, diabetic women have a higher mortality risk than diabetic men. The risk of excess mortality in women appears to be impacted by age. Although several studies corroborate a higher mortality risk in older women, others suggest a higher risk among younger groups of women. Greater inflammation and oxidative stress in diabetic women with end-stage renal disease, as well as gender-related aspects regarding access to treatment and management modalities might, at least partly, explain excess mortality in diabetic women [172]

The prevalence of retinopathy in the diabetic population of the National Health and Nutrition Survey was 28.5%. Male sex, higher glycosylated-hemoglobin levels, longer duration of diabetes, higher blood pressure, and insulin use were associated with the development of retinopathy [170]. The male sex has been reported to be an independent risk factor for advanced diabetic retinopathy and for the progression of the disease. However, the findings are not universal; with several other studies suggesting that the female sex is an independent risk factor for the development of diabetic retinopathy. These controversies are related to a number of factors in the studies, including age (and thus hormone levels), glycemic control, diabetes duration, and ethnic background [169].

Diabetes in men is usually diagnosed at a younger age and with lower body fat mass than in women. Women appear to have a greater burden of risk factors at the time of diagnosis of diabetes, especially obesity. Compared to men, women have a lower risk of macrovascular or microvascular disease in the absence of diabetes. The presence of diabetes confers a higher risk of vascular complications in women compared to men. Some potential explanations include the contribution of sex hormones and specific risk factors for sex. A growing body of evidence suggests that sex hormones play an important role in the regulation of cardiovascular function. In general, estrogens are considered cardioprotective and androgens are detrimental to cardiovascular health. However, recent evidence indicates the diversity and complexity of the action of sex hormones on target tissues, especially in the context of diabetes. Understanding the mechanisms of sex differences in the pathophysiology of diabetic vascular complications may contribute to personalized- and sex-specific prevention and treatment for diabetic macro- and microvascular disease [172].

## 5. Conclusions

In conclusion, women get sick differently than men. Both the symptoms of the same diseases and their courses differ between women and men. In women, most vascular diseases are often atypical and asymptomatic. In addition, the resulting guidelines for the treatment of vascular diseases are based on the results of studies in which the participants were predominantly men. It has detrimental consequences for their medical care. One of the reasons for this was the assumption that the health differences between men and women were largely due only to their reproductive systems [173]. The lack of good epidemiological studies or medical trials in women means that they are diagnosed and treated like men.

It should be noted that men and women respond differently to medications. It may be related to the structure of the atherosclerotic plaque, the vessels’ reactivity, and the endothelium’s functioning, and to the absorption, metabolism, and distribution of chemical substances, including medications.

Furthermore, there are also differences between women and men around the use of guideline-directed medical therapy/optimal medical treatment. Women are less likely to receive statins, antiplatelet agents, and angiotensin-converting enzyme inhibitors than men.

In addition to classic risk factors, such as hypertension, obesity, smoking, lack of physical activity, or abnormal cholesterol levels, women are exposed to additional gender-specific risks associated with changes in pregnancy (hypertension or pregnancy-induced diabetes), as well as hormonal disorders of reproductive age (polycystic ovary syndrome, premature menopause). The presence of an additional burden, from risk factors unique to women, requires intensive measures to prevent the development of vascular diseases and allow their effective treatment. Although most classic risk factors affect both men and women to a similar extent, increasing their chances of developing vascular disease, smoking has been shown to significantly worsen the prognosis in women.

The other issue is women’s knowledge and awareness of vascular diseases. The risk of cardiovascular events is drastically underestimated by women themselves, as well as by those around them. According to German research, more than two-thirds of women surveyed consider breast cancer to be the greatest risk and the most common cause of their fear, but only 25% of women cite cardiovascular disease in this context [174].

In conclusion, to improve the medical care and treatment of women with vascular diseases, the above issues should be considered. Additionally, it is crucial to reinforce the idea related to vascular diseases and the importance of representation in medical associations, among practitioners, and in administrative positions to address the issues associated with women’s diagnosis and treatment effectively. Despite that, it is essential to include the significance of these roles in initiating paradigm shifts within medical education.

## Figures and Tables

**Table 1:** Symptoms of Takayasu Arteritis, according to the vessel involved [102].

Vessel	Symptoms
Carotid arteries	Dizziness, amaurosis fugax, vision loss, transient ischemic attacks, stroke
Subclavian arteries	Arm claudication, inter-arm blood pressure difference >10 mm Hg. Proximal subclavian obstruction can cause subclavian steal
Vertebral arteries	Stenosis/occlusion: vertigo or cerebral ischemic symptoms when multiple arch branches are obstructed
Ascending and arch of aorta	Aortic regurgitation due to aortic dilatation, aneurysm, rarely aorta stenosis
Descending aorta	Systemic hypertension, dyspnea, and heart failure due to aorta stenosis, aneurysm
Abdominal aorta	Systemic hypertension, lower limb claudication, aneurysm
Renal arteries	Systemic hypertension, renal failure
Mesenteric arteries	Postprandial abdominal pain, weight loss, sitophobia, gastrointestinal hemorrhage
Iliac and femoral arteries	Lower limb claudication or fatigue
Coronary arteries	Angina, heart failure, myocardial infarction
Pulmonary arteries	Dyspnea, cough, chest pain, pulmonary hypertension

**Table 2:** The most important risk factors for venous thromboembolism.

Triggering Factors (Acute Conditions)	Predisposing Risk Factors
Surgery	History of VTE
Trauma	Chronic heart failure
Acute internal medicine disease	Older age
Acute heart failure	Varicose veins
Acute respiratory failure	Obesity
Central vein catheter	Immobility/paresis
	Malignancy/myeloproliferative disease
	Pregnancy/peripartum period
	Inheritance vs. acquired thrombophilias
	Hormone treatment
	Renal failure

## Data Availability

No new data were created or analyzed in this study. Data sharing does not apply to this article.

## Abbreviations and Acronyms

AAA	Abdominal aortic aneurysm
AHA	American Heart Association
CAD	Coronary artery disease
CUS	Compression ultrasound
CVD	Cardiovascular disease
CVI	Chronic venous insufficiency
CVRF	Cardiovascular risk factor
DM	Diabetes mellitus
DMARD	Disease-modifying antirheumatic drugs
DVT	Deep vein thrombosis
EHIT	Endovenous heat-induced thrombosis
EM	Early menopause
EVLA	Endovenous Laser Ablation
GDM	Gestational diabetes mellitus
HDP	Hypertensive disorders of pregnancy
HLD	Hyperlipidemia
HTN	Hypertension
IMS	International Menopause Society
LEAD	Lower extremity arterial disease
MHT	Menopausal hormone therapy
MI	Myocardial infarction
PAD	Peripheral arterial disease
PCOS	Polycystic ovary syndrome
PCS	Pelvic congestion syndrome
PE	Pulmonary embolism
PM	Premature menopause
RFA	Radiofrequency ablation
VAD	Venoactive drugs
VSS	Vasospastic syndrome
VTE	Venous thromboembolism
WHI	Women’s Health Initiative
